# *VERNALIZATION1* controls developmental responses of winter wheat under high ambient temperatures

**DOI:** 10.1242/dev.172684

**Published:** 2019-02-15

**Authors:** Laura E. Dixon, Ildiko Karsai, Tibor Kiss, Nikolai M. Adamski, Zhenshan Liu, Yiliang Ding, Vincent Allard, Scott A. Boden, Simon Griffiths

**Affiliations:** 1 Department of Crop Genetics, John Innes Centre, Norwich Research Park, Norwich, NR4 7UH, UK; 2 Department of Molecular Breeding, Centre for Agricultural Research, Hungarian Academy of Sciences, H-2462, Martonvásár, Hungary; 3 Department of Cell and Developmental Biology, John Innes Centre, Norwich Research Park, Norwich, NR4 7UH, UK; 4 Université Clermont Auvergne, INRA, UMR 1095 GDEC (Genetic, Diversity and Ecophysiology of Cereals), 63000 Clermont Ferrand, France

**Keywords:** Flowering, Temperature, Wheat

## Abstract

Low temperatures are required to regulate the transition from vegetative to reproductive growth via a pathway called vernalization. In wheat, vernalization predominantly involves the cold upregulation of the floral activator *VERNALIZATION1* (*VRN1*). Here, we have used an extreme vernalization response, identified through studying ambient temperature responses, to reveal the complexity of temperature inputs into *VRN-A1*, with allelic inter-copy variation at a gene expansion of *VRN-A1* modulating these effects. We find that the repressors of the reproductive transition, *VERNALIZATION2* (*VRN2*) and *ODDSOC2*, are re-activated when plants experience high temperatures during and after vernalization. In addition, this re-activation is regulated by photoperiod for *VRN2* but was independent of photoperiod for *ODDSOC2*. We also find this warm temperature interruption affects flowering time and floret number and is stage specific. This research highlights the important balance between floral activators and repressors in coordinating the response of a plant to temperature, and that the absence of warmth is essential for the completion of vernalization. This knowledge can be used to develop agricultural germplasm with more predictable vernalization responses that will be more resilient to variable growth temperatures.

## Abstract (precis)

**Highlighted Article:** Analysis of the flowering and growth responses of a range of *Triticum aestivum* cultivars to warmer growth temperatures reveals an additional layer of temperature regulation in the vernalization response pathway.

## INTRODUCTION

The ability of plants to sense and respond to changes in the environment is crucial to their survival and reproductive success. Temperature and photoperiod are the principal environmental signals used as seasonal cues by plants to coordinate developmental decisions, including the vegetative-to-reproductive transition. In agriculture, the manipulation of genetic pathways that respond to changes in photoperiod and temperature to initiate flowering has been used to expand geographical cultivation ranges such that flowering and grain/fruit development occurs under optimal conditions (Jung and Müller, 2009). Vernalization is the response to prolonged cold treatment that is required for certain plants to become competent to flower and typically functions by initiating expression of floral activators following cold temperatures, and/or suppressing the activity of floral repressors. In cereals, variant alleles enable a spring growth habit by constitutive expression of floral activators or suppression of the activity of floral repressors, and breeders have used these alleles to expand cultivation into regions that do not experience sufficient periods of cold to promote flowering or where winter temperatures are lethal. The vernalization response is quantitative with increasing duration (weeks) of low temperatures leading to a continued reduction of flowering time until the vernalization requirement is satisfied, as determined by an absence of any earlier flowering with further extensions in duration of cold treatment (Kim et al., 2009). In certain plant species (e.g. rye), the vernalizing effect of prolonged cold temperatures is lost or possibly reversed by a short period of high temperatures (∼35°C), and this effect is referred to as de-vernalization (Gregory and Purvis, 1948). The genes that function during the vernalizing cold treatment are well understood; however, little is known about the genetic regulation of flowering under variable ambient growth temperatures or the molecular nature of the de-vernalization response. It is essential that the molecular response to varying temperatures is understood to enable predictive selection of alleles that will maintain or improve crop yield under warmer and more variable climates.

In bread wheat (*Triticum aestivum*), the MADS-box transcription factor gene *VERNALIZATION1* (*VRN1*) is a floral activator that is central to the regulation of vernalization and its expression is activated under cold temperatures (Alonso-Peral et al., 2011; Danyluk et al., 2003; Trevaskis et al., 2003; Yan et al., 2003). Dominant mutations in *VRN1*, in either the A, B or D genome or in the translocation of *VRN1* to chromosome 5DS (*VRN-D4*), that cause its overexpression, bypass the vernalization requirement and result in a spring habit (Kippes et al., 2015; Fu et al., 2005). The flowering repressor, which is bypassed by high *VRN1* levels, is a grass-specific CCT-domain gene *VERNALIZATION2* (*VRN2)* (Chen and Dubcovsky, 2012; Yan et al., 2004). In winter-habit plants, as day length decreases during autumn, *VRN2* expression drops to reduce *VRN2*-mediated repression of the central flowering activator *FLOWERING LOCUS T1* (*FT1*) (also named *VERNALIZATION3*) (Yan et al., 2006). Short days in combination with low temperatures allow *VRN1* expression, which together with a lengthening photoperiod induces *FT1* expression and promotes floral transition of the meristem (Hemming et al., 2008; Li and Dubcovsky, 2008; Yan et al., 2006). Regulation of both the habit and strength of the vernalization response is observed through regulation of *VRN-A1*. A glycine-rich RNA-binding protein, *GPA2*, has been identified that binds regulatory elements in intron 1 of *VRN1* and leads to the repression of *VRN1* expression (Kippes et al., 2016; Xiao et al., 2014). Additionally, single nucleotide polymorphisms (SNPs) in exons 4 and 7 have been identified that influence the duration of cold temperatures required to complete vernalization and the alleles of *VRN1* have been shown to have different geographical distributions (Eagles et al., 2011; Muterko and Salina, 2018). An exon 4 SNP results in an amino acid change of L117F in the conserved k-domain (Chen et al., 2009; Eagles et al., 2011; Díaz et al., 2012). The effects of this SNP have been investigated in winter wheat containing multiple copies of *VRN-A1*; a cultivar Hereward with two copies of T-type and one C-type vernalized slower than Malacca, which contained one C-type and one T-type (Díaz et al., 2012). Another *VRN-A1* SNP that causes an amino acid substitution, A180V in exon 7, also regulates vernalization duration, via its regulation of a protein interaction with *Ta*HOX1 (Li et al., 2013). Beyond regulation permitted by alterations in nucleotide sequence there is increasing evidence that cereal vernalization is also regulated at the epigenetic level. A study in barley identified that epigenetic regulation of *HvVRN1* was important, and in *Brachypodium* the epigenetic regulation of *BdFT1* influences the progression of vernalization (Oliver et al., 2009; Huan et al., 2018). Further regulation of *HvVRN1* has been identified in response to ambient temperatures where it has been identified to have a role in controlling spikelet number at higher ambient temperatures (Ejaz and von Korff, 2017). This highlights that there may be further species-specific adaptation in the vernalization response.

In this study, we show that there are additional points of temperature control in the vernalization network beyond cold regulation of *VRN1* and that signalling effects of warmer temperatures need to be understood to optimize vernalization alleles to environments with variable climates.

## RESULTS

### Vernalization is a response to a wide range of temperatures

**Fig. 1.**
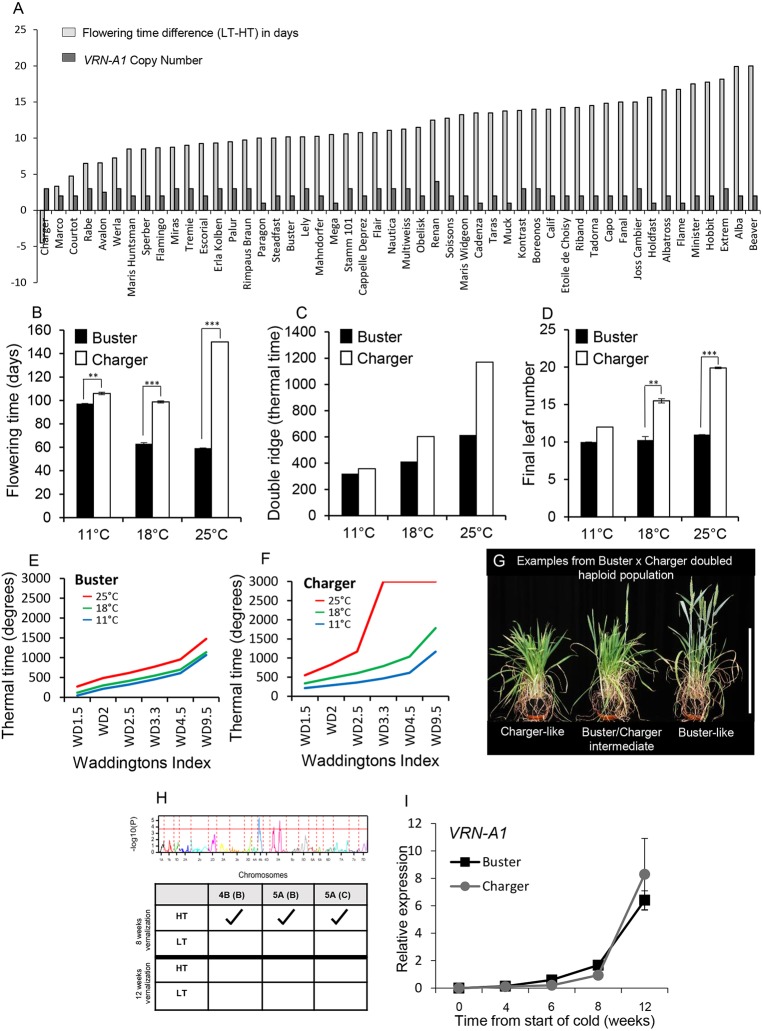
**A high ambient temperature developmental delay genetically maps near to *VRN-1A*.** (A) Difference in flowering time in days between LT (18°C: 13°C) and HT (24°C: 19°C) for winter wheat cultivars (light grey bars) and *VRN-A1* haploid CN (dark grey bars). (B-D) Flowering time (B), thermal time to double ridge (C) and final leaf number (D) for Buster and Charger at constant 11°C, 18°C or 25°C. *n*=4. Error bars represent s.e.m. (E,F) Time course of the primary apex under constant 11°C, 18°C or 25°C for Buster (E) and Charger (F) (where Charger did not reach certain developmental stages the result is shown as 3000). (G) Representative images of genotype habits following 8 weeks of vernalization and growth at HT. Scale bar: 40 cm. (H) QTL analysis of B×C population following 8 weeks of vernalization and then growth under either LT or HT. The table summarizes significant QTL peaks identified from growth under LT or HT following 8 or 12 weeks of vernalization. (I) *VRN-A1* gene expression in Buster and Charger over a vernalization time course. *n*=3 biological replicates, each formed of three leaf tissue samples from independent plants. Error bars represent s.e.m. Expression normalized as described in Materials and Methods. **P<0.01, ***P<0.001 determined by paired Student's *t*-test. Significance not determined at 11°C for final leaf number (C) as there was no variance in the measurements taken.

To identify how ambient temperature regulates flowering time in bread wheat (*Triticum aestivum*) and to assess genetic variation for developmental responses at different growth temperatures, we measured flowering time in 98 cultivars (Table S1) that had been vernalized for 8 weeks (45 days) and then grown under two temperature conditions: 18°C in the light, 13°C in the dark (referred to as low temperature, LT), and 24°C in the light, 19°C in the dark (high temperature, HT), under long days (LD). The range in flowering time between the temperatures was 26.5 days where, with one exception, HT led to earlier flowering (Fig. 1A, Fig. S1). The single exception was the cultivar Charger, which flowered earlier under LT. To validate this result and identify the developmental stage when temperature sensitivity occurred, Charger was compared with a genetically similar (winter habit, photoperiod sensitive) wheat, Buster, which displayed an intermediate temperature response. We measured the time to spike emergence (flowering time), timing of the vegetative-to-reproductive transition (double ridge) and final leaf number of plants grown under LD with constant temperatures of 11°C, 18°C or 25°C (Fig. 1B-D). Consistent with the previous experiment (Fig. 1A), flowering was delayed in Charger at 25°C, relative to 11°C and 18°C, whereas warmer temperatures accelerated flowering in Buster. We also observed that Charger exhibited a delay in the vegetative-to-reproductive transition [double ridge; 357 in thermal time (TT) at 11°C versus 1169 TT at 25°C] and an increased production of leaves under warmer growth temperatures. In comparison, the delay in transition of Buster was only 36% of that observed in Charger, and there was no significant effect on leaf number (Fig. 1C,D). Detailed analysis of the vegetative-to-floral transition apex stages identified that the early stages of inflorescence development could be completed by Charger at 25°C but terminal spikelet was generally not reached (Fig. 1E,F).

As Buster and Charger showed differential flowering responses to HT, we developed a Buster×Charger (B×C) doubled haploid population to identify the controlling genetic region(s) (genetic map is provided in Table S2). Under LT, all lines flowered. At HT, the Charger-like behaviour was observed to segregate across the population with five lines not flowering within the duration of the experiment (Fig. 1G, Fig. S2). Quantitative trait locus (QTL) analysis identified three significant QTLs; however, only one region on chromosome 5A had the late-flowering allele from Charger (Fig. 1H, Table S2). The QTL region contained *VRN-A1* (Fig. S3); however, the markers used to develop the genetic map were monomorphic for *VRN-A1* between Buster and Charger which would prevent this gene from being identified as the QTL peak. We hypothesized that, given the position of the QTL and that the response was temperature related, the vernalization requirement for Charger was not satisfied and that LT but not HT was sufficient to meet and sustain the vernalization requirement, even though LT was much higher than ‘standard’ vernalizing temperatures. To test this hypothesis, the B×C population was vernalized for 12 weeks at 6°C under short days (SD), a duration that exceeds previous reports for vernalization requirement (Berry et al., 1980), and then grown under the HT and LT regimes. All lines flowered and the 5A QTL was not detected (Fig. 1H, Fig. S4), suggesting that vernalization had been incomplete under the standard vernalization treatment used in the screen and that the LT had enabled its completion. To confirm that this response was temperature mediated, as opposed to the possibility that increased duration under SDs had a vernalizing effect, Buster and Charger were vernalized at 6°C under either LD or SD photoperiods for 12 weeks. Both genotypes flowered comparably following both conditions, highlighting that photoperiod was not driving the delay in flowering at HT (Fig. S5). This further supports that the QTL is underpinned by *VRN-A1* rather than the relatively closely situated gene *PHYTOCHROME C* (*PHYC*); however, it does not exclude a role for *PHYC* in ambient temperature responses in wheat.

### Different *VRN-A1* haplotypes found within the same genotype show distinct regulation and link with floral timing

**Fig. 2.**
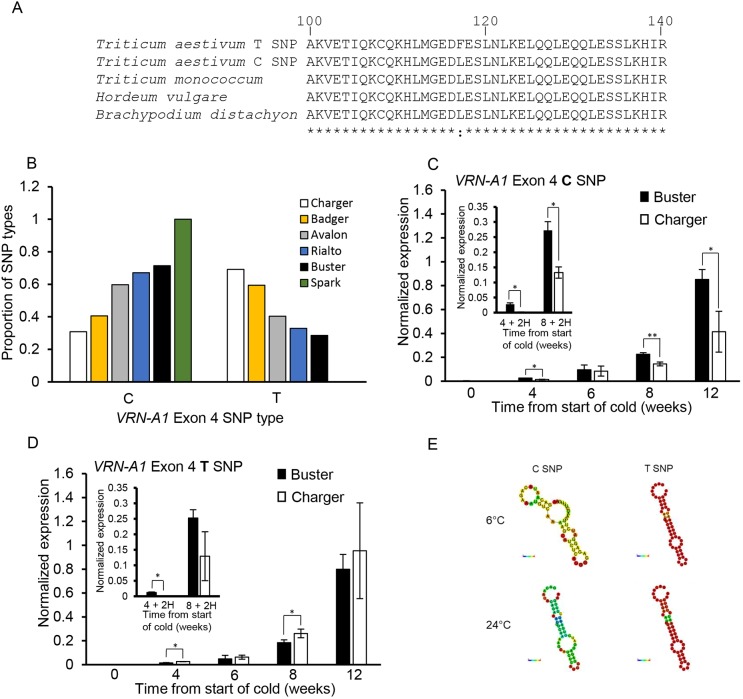
***VRN-A1* haplotypes are differentially regulated within the same genotype.** (A) Protein sequence alignment highlighting the conserved location of the C/T SNP. (B) The proportion of C- and T-type alleles in exon 4 of *VRN-A1* determined through exome-capture sequencing of wheat cultivars Buster, Charger, Avalon, Badger, Spark and Rialto. (C,D) Expression of Buster and Charger across a 12 week vernalization time course with inset showing expression of Buster and Charger following 4 or 8 weeks of vernalization and 2 weeks at 24°C (2H) for *VRN-A1* exon 4 C-type (C) and *VRN-A1* exon 4 T-type (D) alleles. (E) mRNA secondary structure predictions for the *VRN-A1* region containing either the exon 4 C- or T-type allele at 6°C or 24°C. Vernalization samples in C and D are the same samples as used in Fig. 1I. *P<0.05, **P<0.01, determined by paired Student's *t*-test.

The QTL position and vernalization response suggested that Charger might carry a novel allele of *VRN-A1* that influences vernalization and flowering under warm temperatures, as variation in *VRN-A1* has previously been reported to influence the duration of vernalization (Table S3). To test this, we interrogated the *VRN-A1* alleles of Charger and Buster. Sanger sequencing did not detect differences for *VRN-A1* between Buster and Charger, which contain alleles for long vernalization winter habit (Table S3; Chen et al., 2009; Díaz et al., 2012). In addition, expression of *VRN-A1* was very similar in each cultivar when measured over a standard vernalization time course at constant 6°C (Fig. 1I). A further source of variation in vernalization response in wheat occurs via copy number (CN) of *VRN-A1* (Díaz et al., 2012). We quantified *VRN-A1* CN of Charger and Buster using a Taqman assay and found that Charger contains three haploid copies whereas Buster has two copies (Table S4). This suggested that CN of *VRN-A1* could be the causal difference. To test this hypothesis, we assessed whether *VRN-A1* CN correlated with the temperature response observed in our screen (Fig. 1A). A total of 18 out of 50 wheat cultivars with a winter habit also contained three or more haploid copies of *VRN-A1*, with multiple genotypes containing the same long-winter allele found in Buster and Charger (Fig. 1A, Table S4, Fig. S6). There was no significant difference in average flowering time under LT or HT conditions between cultivars with two or three *VRN-A1* haploid copies. The difference in flowering time between LT and HT was also not significant between two and three copies of *VRN-A1* (Fig. S6). This indicates that CN was not regulating the extended vernalization duration observed in Charger. However, previous research had demonstrated that *VRN-A1* CN did influence vernalization duration and identified different haplotypes of *VRN-A1*, where multi-copy genotypes contain combinations of two *VRN-A1* alleles: a wild-type (WT) C-type *VRN-A1* allele and an alternate T-type allele, defined by a C/T SNP located in exon 4 (Díaz et al., 2012). This SNP invokes a missense mutation of a conserved residue (Díaz et al., 2012) (Fig. 2A) by substituting leucine with a phenylalanine at position 117 (C10428T; L117F), which is predicted to be a deleterious substitution (−3.971 PROVEAN score; Choi et al., 2012). However, although the protein encoded by the T-type SNP may be less active or lack function, the observed phenotypic differences suggest that the allele still contributes to the vernalization response, which is supported in multi-copy genotypes that display different vernalization requirements and contain the same number of C-type but differ in the number of T-type *VRN-A1* genes (Díaz et al., 2012). We hypothesized that altered proportions of these alleles could influence the response to warm temperatures. To identify the allelic compositions at the *VRN-A1* locus in Buster and Charger, we conducted exome-capture sequencing, which detected differing ratios of the C-type allele (WT) relative to the T-type (mutant) allele. Charger contained a higher frequency of the mutant T-type allele (0.7 T-type in Charger versus 0.28 in Buster; Fig. 2B), which has been reported to have a slower rate of induction than the C-type allele (Díaz et al., 2012). To test whether the proportion of C/T alleles in exon 4 is linked with the vernalization response, we examined exome-capture data for a subset of phenotypically distinct cultivars (Fig. 2B). The cultivar Badger has a Charger-like flowering response and contained the second highest frequency of T-type allele (0.6 T-type to 0.4 C-type). The remaining cultivars showed a lower frequency of T-type allele (0.4 Avalon, 0.32 Rialto), which correlated with a Buster-like flowering phenotype when vernalization was interrupted by warm temperatures (for comparison refer to Fig. 4C). This assessment was also supported through analysis of Buster×Charger doubled haploid lines, which represented the two flowering phenotypes (flowering, or not, under high temperatures) where the lines that did not flower under high temperatures contained three haploid copies of *VRN-A1* and were Charger-like in phenotype, whereas plants that displayed a Buster-like phenotype contained only two haploid copies of *VRN-A1* (Table S4C)*.*

To investigate how the proportion of C and T-type alleles in exon 4 could be responsible for the long/warm temperature vernalization phenotype, we performed allele-specific expression analysis (Díaz et al., 2012). Transcript levels of the T-type allele were very similar in Buster and Charger, whereas the C-type showed differing levels after 8 weeks of vernalization (Fig. 2C,D). This suggested that the C-type allele has a more crucial role for completing and maintaining vernalization than the T-type and that the expression of *VRN-A1* C-type could be in part regulated by the cumulative CN of *VRN-A1.* Both Buster and Charger contain one copy of the C-type allele and then one (Buster) or two (Charger) copies of the T-type, which is proposed to encode a less functional protein, yet Buster expresses more *VRN-A1* C-type consistently through vernalization (Fig. 2C). To test expression differences further, we measured transcript levels for the *VRN-A1* alleles under the conditions of 8 weeks vernalization followed by 2 weeks high temperature, which is when the response in vernalization is phenotypically distinguishable (Fig. 2). We observed that transcript levels increased for both SNP types with increasing cold and that the C-type SNP was significantly higher in Buster under constant cold and following the warm temperature pulse than Charger. The T-type transcript pattern was similar to the C-type but, notably, the relative expression between the genotypes altered such that Charger expressed less T-type SNP than Buster following the warm pulse, whereas under continuous low-vernalizing temperatures, Charger expressed slightly higher levels of T-type SNP, relative to Buster (Fig. 2C,D, inset). These results indicate that the expression of the C-type allele influences flowering under warmer temperatures more strongly than the T-type allele but also that the reduction in expression of the T-type allele in Charger under warmer temperatures does contribute to flowering regulation. The reduced contribution of the T-type allele may be due to the missense mutation reducing the floral promoting activity of *VRN1*, or alternatively perturbing its ability to suppress expression of *VRN2* (the floral repressor) upon return of plants to warm ambient temperatures (Deng et al., 2015). *In silico*, computational prediction suggests that the T-type allele results in a more stable secondary structure of the transcribed *VRN-A1* RNA relative to the C-type allele both at 6°C (ΔG −21.10 kcal/mol and ΔG −16.86 kcal/mol for the T- and C-type allele, respectively) and 24°C (ΔG −14.57 kcal/mol and ΔG −10.54 kcal/mol for the T- and C-type allele, respectively) (Fig. 2E). The greater stability of the secondary structure at 6°C and 24°C may contribute to the reduced influence of the T-type allele on the vernalization response owing to perturbed RNA stability, anti-sense function, or translational efficiency, relative to the C-type allele.

### Sustained expression of floral activators and suppression of repressors is required for flowering under warm ambient temperatures

**Fig. 3.**
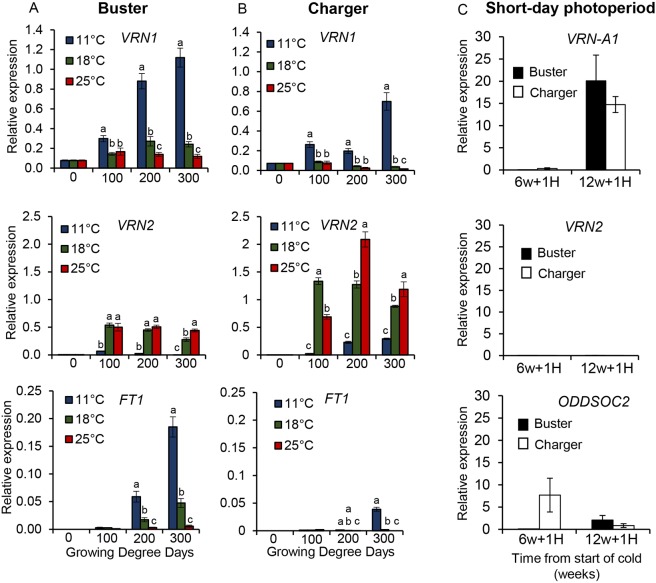
**Warm temperatures and long days promote the expression of the floral repressor *VRN2* during and after vernalization.** (A,B) The relative expression of *VRN1*, *VRN2* and *FT1* at 0, 100, 200 and 300 growing degree days (GDD) following vernalization (45 days at 3°C) and then growth under post-vernalization treatments of either constant 11°C (blue), 18°C (green) or 25°C (red) LDs for Buster (A) and Charger (B). Significance in A and B is shown relative to 0 GDD and determined by one-way ANOVA. Inter-cultivar comparison can be made as the 0 GDD levels are extremely similar; *VRN1*: 0.078 Buster and 0.073 Charger; *VRN2*: 0.004 Buster and 0.005 Charger; and *FT1*: 0 Buster and 0 Charger. (C) The relative expression of *VRN-A1*, *VRN2* and *ODDSOC2* following 6 or 12 weeks of vernalization (SD at 6°C) and 1 week of 24°C under SD. *n*=3 biological replicates, with sampling and normalization described in Materials and Methods. Error bars represent s.e.m.

Our current understanding of vernalization in wheat is that cold temperatures facilitate transcriptional activation of *VRN1* (the floral activator), which enables *VRN2* (the floral repressor) to be repressed. However, the different expression levels in *VRN-A1* C- and T-type alleles following a warm temperature treatment (Fig. 2) are consistent with the possibility that the transcriptional response of *VRN1* to warm temperatures is also important in determining the required duration of vernalization. To investigate whether warm temperature signalling influences other components in the vernalization network, we measured the expression of *VRN2* following standard vernalization (45 days at 3°C) and transfer into 11°C, 18°C or 25°C. Under these conditions, vernalization of Buster was saturated but the Charger requirement was not, suggesting that the vernalization response of Charger would still respond to warmer temperatures. Unexpectedly, *VRN2* expression was significantly increased under warmer temperatures of 25°C and 18°C, relative to 11°C, in both genotypes (Fig. 3). As anticipated, *VRN1* levels were lower at 25°C and 18°C than at 11°C (Fig. 3) (Alonso-Peral et al., 2011; Chen and Dubcovsky, 2012; Yan et al., 2003). *FT1* expression was also lower at 25°C and 18°C than 11°C (Fig. 3). The degree of re-activation of *VRN2* expression was higher in Charger than Buster but, notably, the re-activation of *VRN2* expression in Buster occurred even when vernalization was fully satisfied, suggesting that this aspect is not part of the molecular basis observed in de-vernalization (Gregory and Purvis, 1948). *VRN2* is also regulated by photoperiod, as it is activated by LD (Dubcovsky et al., 2006). To investigate involvement of the LD induction of *VRN2* expression for the observed flowering phenotypes, we interrupted vernalization after 6 weeks (when both genotypes have incomplete vernalization) or after 12 weeks (when vernalization is satisfied for both genotypes) with 1 week at 24°C under SD photoperiod. Expression was measured for *VRN-A1*, *VRN2* and *ODDSOC2*, a MADS-box gene that has been shown to be sensitive to temperature (Hemming et al., 2012; Trevaskis et al., 2003). Under SDs *VRN2* was not expressed, and *ODDSOC2* expression was identified to be temperature but not photoperiod dependent (Fig. 3C). This suggests that part of the regulation of *VRN2* expression is mediated through photoperiod, as well as temperature, and supports the observation that *VRN1* has a crucial role in maintaining *VRN2* repression after vernalization is complete (Chen and Dubcovsky, 2012; Deng et al., 2015). These expression data indicate that the differential response of Buster and Charger to warmer ambient temperatures involves continued transcriptional regulation of the floral activator (*VRN1*) and repressor (*VRN2*) under variable temperatures. In addition, linked with Charger's requirement for a longer duration of cold to vernalize sufficiently, Charger also displays stronger re-activation of the repressor, relative to Buster.

### Understanding the role of warmer temperatures in vernalization is essential to combat the genotype versus environment mismatch

The re-activation of *VRN2* and *ODDSOC2*, along with the differing expression rates of the two *VRN-A1* haplotypes at different temperatures shows that vernalization is not just a response to the duration of cold, but also to the absence of warmth. This highlights a very important aspect of vernalization that has been poorly considered, which involves the dynamic response of activators and repressors in controlling the duration of vernalization under variable temperature conditions. Our results suggest that genotypic variation in the response to warm temperatures should also be clearly distinguishable, much as it is for the duration of vernalization under constant cold conditions.

**Fig. 4.**
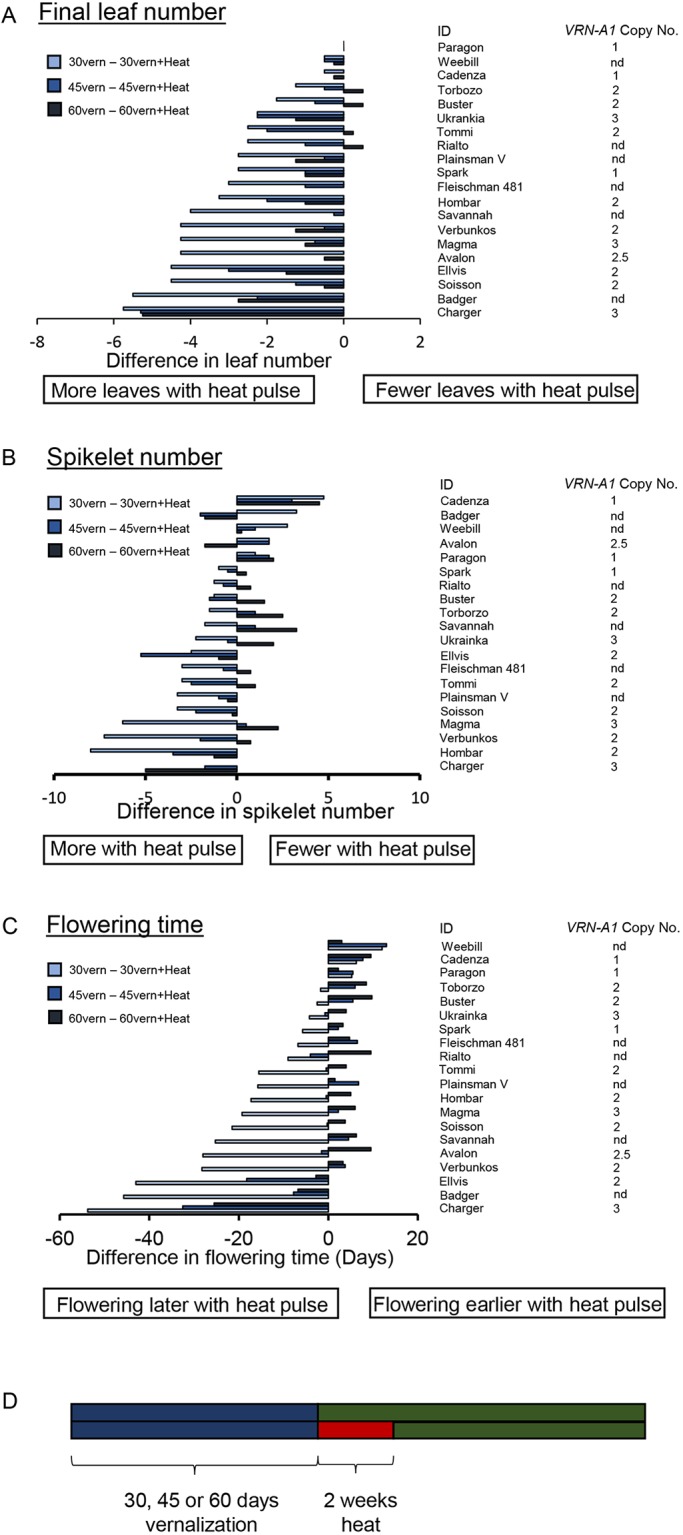
**Vernalization shows that stage-specific responses to both cold and absence of cold do not directly correlate with *VRN-A1* CN.** (A-C) The differences between high-temperature treatment (2 weeks at 24°C, LD) and control without the heat treatment, following three vernalization (at 3°C) conditions; 30 days (light blue), 45 days (medium blue) and 60 days (dark blue) for final leaf number (A), spikelet number (B) and flowering time (C). (D) Schematic of the experimental conditions. nd, copy number not determined.

To examine the extent of the genotypic diversity of the vernalization response to variable temperatures, we tested a selection of wheat cultivars, which included spring cultivars (Weebill, Cadenza, Paragon) and cultivars containing one, two or three copies of *VRN-A1*. Plants were vernalized for 30, 45 or 60 days and then transferred for 2 weeks to 25°C before being moved to the glasshouse, or moved directly from the vernalization treatment to glasshouse. The analysis of the final leaf number, spikelet number and flowering time (Fig. 4A-C) indicated that there is diversity in the response to warm temperatures experienced during vernalization. Additionally, our analysis shows that vernalization does not progress in a linear fashion such that a significant delay could be observed after 30 days of vernalization and warm temperature pulse but only a very minor delay following 45 days vernalization and warm temperature pulse, relative to control conditions with no warm temperature pulse. Altering the duration of vernalization via a warm temperature break directly influenced spikelet number (Fig. 4B), which indicates a delay during early stages of inflorescence development. This is supported by our observation that at 25°C the transition of the inflorescence apex in Charger is delayed at all stages, usually not completing terminal spikelet, whereas in Buster, where vernalization is complete, there is only a slight delay in the rate of apex transition at 25°C (Fig. 1E). In spring cultivars, the warm temperature break always accelerated growth and resulted in fewer spikelets (Fig. 4B). In winter cultivars, the largest increase in spikelet number was observed following the temperature break after 30 days of vernalization. However, again it was not possible to predict the response using *VRN-A1* CN. Flowering time was strongly regulated by the high temperature break with Charger showing the most significant delay, but Badger and Ellvis also exhibited a flowering delay of over 40 days following the warm temperature break after 30 days vernalization. This re-iterates that the response observed in Charger is an extreme version of that found in winter cultivars and that the variation in the response to warmer temperatures may be prevalent and important in regulating the duration of vernalization required by winter wheat.

## DISCUSSION

### Interrogating ambient temperature responses uncovered a broader temperature range in the vernalization response

In this study, we screened a diverse panel of hexaploid wheat cultivars under ambient temperatures that are relevant to multiple mega-environments, to characterize a molecular response of winter wheat to warmer growth temperatures. Our analysis has identified an additional layer of temperature regulation in the vernalization response pathway. Vernalization is a quantitative response. In vernalization-requiring plants (winter habit), increasing the duration (weeks) of low temperatures early in development leads to increasingly early flowering, until the vernalization requirement has been saturated (Kim et al., 2009). We observe that under constant low temperature conditions the molecular vernalization response, as determined by *VRN1* expression, is very similar between two photoperiod-sensitive winter wheat cultivars, Buster and Charger (Fig. 1I). However, when constant cold temperatures are interrupted or ended with a higher temperature the vernalization profile, and flowering time, are significantly different between genotypes, with Charger showing a delay in flowering time (Fig. 1A, Fig. 2). This highlights that vernalization actively responds to higher temperatures, possibly via the de-vernalization response described by Gregory and Purvis (1948). However, our analysis shows that the response to warmer temperatures is not simply through delayed vernalization, as vernalization can proceed under much warmer temperatures than those classically considered to be vernalizing, >6°C (Chouard, 1960), with Charger completing vernalization at temperatures in the range 13-18°C (Fig. 1). This result is consistent with previous reports that showed warmer temperatures were vernalizing in wheat and carrot (Yan and Hunt, 1999). Recently, warmer and more variable temperatures have been shown to influence vernalization in *Arabidopsis thaliana* (Duncan et al., 2015; Hepworth et al., 2018). Based on these observations, together with our results, we propose that the thermal-dependent regulation of flowering in wheat involves a responsive interaction between the plant and its environment to coordinate the expression of floral activators and repressors over a range of temperatures.

### Multi-copies of *VRN-A1* with different haplotypes show altered temperature responsiveness

We identified a region on chromosome 5A that underpinned the high temperature response. The location on chromosome 5A, supported by phenotypic analysis, suggested that *VRN-A1* was the causal gene. More detailed analysis of the molecular nature of the warm temperature response in Charger indicates that although CN of *VRN-A1* is important for determining the vernalization duration under constant low temperature conditions, the allelic composition of these copies is important in understanding how vernalization will proceed under variable temperatures (Fig. 2) (Díaz et al., 2012). This is further supported by previous reports that *VRN1* expression is responsive to changes in temperature conditions following vernalization (Greenup et al., 2011). We observe expression differences between the C- and T-type SNPs in exon 4 of the *VRN-A1* genes, suggesting that this exon has additional functions in the regulation of *VRN-A1* expression. This could be caused by increased nascent RNA stability, as *in silico* predictions show that the T-SNP has significantly higher stability than the C-type across the temperature range of 6-24°C. Our analysis indicates that the level of C-type allele expression of *VRN-A1* is crucial in determining the vernalization response, as low expression in Charger is associated with late flowering under warm temperatures, relative to Buster, which flowers earlier and has increased expression of the C-type *VRN-A1* allele. We propose that the composition of C-type and T-type alleles of *VRN-A1* is crucial for fine-tuning this response, and that further investigation of this pathway is vital for determining the response of modern cultivars to warmer winter temperatures. It is essential to now develop near isogenic lines carrying different frequencies of *VRN-A1* exon 4 C and T-SNP alleles to empirically confirm and further understand this pathway.

### Additional temperature inputs to vernalization-related genes regulate development pre- and post-vernalization

We observed that the *VRN-A1* locus is regulated by a range of temperatures (6-24°C), which raised the question of whether vernalization response genes also respond to a wider range of temperatures. We observed that *VRN2* expression is activated during and post-vernalization by warm temperatures, in conjunction with LD photoperiod (Fig. 3). This highlights that the vernalization-mediated repression of *VRN2* is not comparable to that observed for floral repressors in other plant species, where the repressor is silenced epigenetically and remains suppressed under subsequent warm temperatures until reset for the next generation (Berry and Dean, 2015). The non-permanent repression of *VRN2* is supported by reports showing that *VRN1* (barley; Oliver et al., 2009) and *FT1* (*Brachypodium*; Huan et al., 2018) are epigenetically regulated during vernalization in grasses. The expression of *VRN2* during and post-vernalization may be linked to different developmental processes, including *VRN1*-dependent suppression of transcription (Deng et al., 2015). During vernalization, *VRN2* may be antagonizing the rise of *VRN1* expression to enable an important delay in vernalization when environmental temperatures are warm; this would fit well with the concept of de-vernalization (Gregory and Purvis, 1948) and offer a mechanism for continued, dynamic and responsive regulation. Following vernalization, *VRN2* may have a role in regulating the procession through the apex transition stages. In agreement with previous studies, we observed that *FT1* did not appear to be directly temperature responsive but rather followed the pattern of expression observed in *VRN1*. This is further supported by *FT1* being a downstream integrator of *VRN1* signalling (Hemming et al., 2012; Dixon et al., 2018).

To understand how ending vernalization with warm temperatures influenced yield potential traits, cultivars were vernalized (30, 45 or 60 days) and then were transferred to standard ambient temperature growing conditions or to 25°C for 2 weeks (Fig. 4). We observed that warm temperatures caused a greater delay in flowering time than the delay caused by incomplete vernalization, which is consistent with previous analysis of *VRN1* in barley under different environmental conditions (Greenup et al., 2011). This phenotypic response is similar to the de-vernalization response identified by Gregory and Purvis (1948). Our molecular analysis suggests that a possible mechanism for this response could be mediated through the re-activation in expression of the floral repressors *VRN2* and *ODDSOC2* under warm temperatures. In addition, we observed that warm temperature caused an early developmental delay (Fig. 1), and this resulted in the formation of additional spikelets. The additional spikelets were only observed in winter genotypes when vernalization was incomplete (Fig. 4). This observation is consistent with reports in barley (Ejaz and von Korff, 2017). An interesting consideration in understanding the molecular nature of this response is that the spring cultivars do not show the warm temperature-mediated delay early in development. As spring habit is determined through the continued high expression of *VRN1*, our results suggest that pre-translational regulation of *VRN1* modulates the temperature response. This observation, in combination with our detection of the thermal response being linked to the exon 4 SNP, suggests a more intricate level of *VRN1* gene regulation than direct control from the promoter. Understanding the molecular mechanism of how early developmental delay is regulated is an important consideration, especially given that the likely increased incidence of unseasonal warm weather will disrupt vernalization delay flowering significantly in winter wheat, which would have dramatically detrimental effects on grain production.

In conclusion, the coordination of plant development with the environment helps to optimize reproductive fitness and yield potential. Vernalization is an essential aspect of this mechanism in winter habit plants. Our research has identified that warm temperatures have an important role, both regarding the temperatures at which vernalization can proceed as well as the re-activation of repressors during and after cold treatment. We show that our current understanding of vernalization is limited by studying the response under constant laboratory conditions. To understand and apply the vernalization response in agriculture, we must dissect the nature of the vernalization pathway under variable and field conditions.

## MATERIALS AND METHODS

### Temperature-response screen

The screen included 98 hexaploid wheat cultivars that are part of two SNP- and simple sequence repeat-genotyped ‘core sets’ from the Watkins and Gediflux collections (Table S1) (Wingen et al., 2014). Additional genotypes included were: *Rht3*, *−10* and *-D1b* near isogenic lines (NILs) in the Mercia background (Pearce et al., 2011) and a *ft-b1* null, line #74 (Dixon et al., 2018). Seeds were germinated at room temperature and the seedlings were vernalized at 3°C under SD photoperiod (8 h light: 16 h dark), low light intensity [12-13 µmol m^−2^ s^−1^ photosynthetic photon flux density (PPFD)] for 45 days. After vernalization, two plants of the same genotype (at the one-leaf stage) were grown in pots of 11 cm (radius)×17 cm (height) filled with a 2:1:1 mixture of garden soil, humus and sand. For each genotype, two plants×two pots were grown in controlled environment chambers (CONVIRON PGB-96 type) set to either HT (24°C during the light: 19°C during the dark) or LT (18°C during the light: 13°C during the dark), both under LD photoperiod (16 h light: 8 h dark). The light source was metal halide lamps and humidity was maintained at 70%.

### Developmental assay

Buster and Charger seeds were germinated and vernalized as described for the screen. Four plants were grown in two pots per genotype, under constant temperature conditions of either 25°C, 18°C or 11°C using the same lighting and humidity conditions described above. Leaf number, double ridge and flowering time were recorded, and the cumulative thermal time calculated. When a given developmental phase was not reached an arbitrary standard high value of 3000 GDD was given to allow statistical analysis.

### Long-day, cold vernalization

Seeds were germinated to coleoptile lengths of 1-3 cm and then grown under long-day, cold vernalization (16 h light: 8 h dark; constant 6°C) for 12 weeks in Snijder Micro-Clime controlled growth chambers. Plants were vernalized in cereals mix soil in trays containing 40 wells (each well 4 cm^2^, 5 cm deep). Following vernalization, the plants were transferred to plastic pots of 7 cm^2^, depth 7 cm, in cereals mix compost and then grown under long-days and LT (18°C light: 13°C dark) or HT (24°C light: 19°C dark) conditions.

### Copy number assay

The TaqMan copy number assay (Díaz et al., 2012) was used to assess the number of genomic copies of the *VRN-A1* gene.

### Population development, genetic mapping and QTL analysis

The Buster×Charger (B×C) doubled haploid population used a maximum of 94 individual lines. JIC1: the B×C population, and parents Buster and Charger, were germinated and then transplanted into 96-well trays (each well 3.5 cm^2^ and 7 cm deep) in cereal mix. Plants were grown to coleoptile lengths of 3 cm and then transferred to vernalization conditions of SD (8 h light: 16 h dark; constant 6°C) for 8 weeks. Plants were then transferred to 7 cm^2^ and 7-cm-deep plastic pots. Three replicates for each genotype were moved into Conviron Controlled Environment Rooms (CERs) at either HT (24°C light: 19°C dark) or LT (18°C light: 13°C dark) under LD photoperiod (16 h light: 8 h dark). Days to flowering, measured by half-ear emergence, was recorded. JIC2: the B×C population with parents Buster and Charger, were germinated as described in JIC1 and transferred to vernalization conditions of SD (8 h light: 16 h dark; constant 6°C) for 12 weeks. Then were treated as described for JIC1.

The B×C population was genotyped using the iSelect 90k array (Trait analysis, Gatersleben, Germany). Following the removal of redundant markers, the map was formed of 695 markers distributed across all chromosomes, with lower marker density on the D-genome (Table S2). QTL analysis was conducted in GeneStat (16th edition) using the single trait linkage analysis (multi-environment) option. Where more than one significant QTL was identified, all were used as co-factors to refine further the identification of stable QTLs. Where the analysis involved individual plants that did not flower within the duration of the experiment, an artificially late flowering time was given to allow QTL analysis. To identify the genes within the QTL region, the B×C genetic map for the 5A chromosome was aligned with the genetic map of the homologous region from the Avalon×Cadenza (A×C) map.

### Sequencing of VRN-A1 and exome-capture sequencing

Sanger sequencing (ThermoFisher) of *VRN-A1* was conducted using primers from Díaz et al. (2012) on DNA extracted from leaf tissue. Exome capture, using the capture array described previously (Krasileva et al., 2017), was conducted by the Earlham Institute, UK, for *Triticum aestivum* cultivars Buster, Charger, Badger, Spark, Rialto and Avalon on genomic DNA extracted from leaf tissue. The raw fasta data of each sequencing run was individually aligned to the IWGSC Refseqv1.0 genomic assembly using BWA-MEM algorithm (version 0.7.12) (Li and Durbin, 2009) with the -M option to mark shorter split hits as secondary hits (for Picard Tools compatibility) and -t to allow multi-threading. The SAM files were formatted into BAM files and sorted using SAMtools (version 1.3) (Li et al., 2009). Optical and PCR duplicates were marked using the MarkDuplicates algorithm from Picard Tools (version 1.134) (http://broadinstitute.github.io/picard) with validation_stringency set to lenient. The resulting BAM files were merged for each genotype (e.g. Avalon) using SAMtools merge. SNPs were called on all genotypes using *FreeBayes* (version 1.1.0) (Garrison and Marth, 2012 preprint) with standard filters (--standard-filters); by default, *FreeBayes* ignores reads that are marked as duplicates. The VCF file was compressed and indexed using Tabix (version 0.2.6) (Li, 2011b); relevant information (chromosome, position, reference allele, alternative allele, etc.) was extracted from the compressed file using BCFtools query (version 1.3.1) (Li, 2011a). Exome-capture data used for this analysis is available on the European Nucleotide Archive under accession number PRJEB30905.

### Gene expression analysis

For the data shown in Figs 1 and 2, plants were grown to coleoptile lengths of around 3 cm and then transferred to vernalization conditions of SD (8 h light: 16 h dark; constant 6°C) for 12 weeks. Leaf tissue was sampled from three plants. For the vernalization time course, samples were taken before transfer to vernalization (time point 0) and then 4, 6, 8 and 12 weeks following the transfer to vernalization. For the LD temperature break, leaf tissue samples were taken after 4 weeks of vernalization followed by 2 weeks at 24°C and 8 weeks vernalization followed by 2 weeks at 24°C. For the SD temperature break, leaf tissue samples were taken after 6 weeks of vernalization followed by 2 weeks at 24°C and 12 weeks vernalization followed by 2 weeks at 24°C. RNA was extracted using the Sigma Spectrum Plant RNA extraction kit and DNase (Promega) treated before cDNA was synthesized using the Superscript II Invitrogen kit (according to manufacturer's instructions) and oligo dT primers. The cDNA was diluted (1:10) and quantitative real-time PCR was conducted using primers developed by Shaw et al. (2013) and normalized to TRIAE_CS42_6DS_TGACv1_544038_AA1746170 (Borrill et al., 2016) with data expressed as a product of 2^^−ΔCT^.

For the data shown in Fig. 3, plants were grown to coleoptile lengths of around 3 cm and then transferred to vernalization conditions of SD (9 h light: 15 h dark; constant 3°C) for 45 days. At the end of the vernalization treatment the plants were at the 1- to 2-leaf stage. They were then transplanted into pots 12 cm in diameter and 18 cm in height, with a soil capacity of 1.5 kg filled with a 4:1 mixture of garden soil and sand, and they were placed into the growth chambers under LD (16 h light: 8 h dark) at three constant ambient temperature levels (11°C, 18°C and 25°C). Leaf tissue was collected at 0, 100, 200 and 300 thermal times (°Cd) after the end of the vernalization treatment. Leaves of three plants were pooled for one biological replicate, with a total of three biological replicates. Total RNA was isolated using the Qiagen RNeasy plant mini kit after Trizol extraction, with an extra step of DNase treatment programmed in the QIAcube equipment (Qiagen). The cDNA transcription was performed from 1 μg of total RNA using the RevertAid First Strand cDNA synthesis kit (Thermo Scientific). Quantitative real-time PCR was carried out with three biological and two technical replicates in a Rotor-Gene Q equipment (Qiagen) applying the SYBR-Green technology of the company. Expression was normalized to Actin using the Rotor-Gene software, which also takes amplification efficiency into account.

### Temperature-break developmental assay

Seeds were germinated and vernalized under the conditions described in the screen. Following 30, 45 or 60 days vernalization plants were either moved to LD (16 h light: 8 h dark) photoperiod conditions for 2 weeks at 25°C in CONVIRON PGB-96 controlled environment chambers or moved directly to the glasshouse. Flowering time, spikelet number and leaf number were measured.

## Supplementary Material

Supplementary information
