# Supplementary material for: VERNALIZATION1 controls developmental responses of winter wheat under high ambient temperatures
Source: Development. 2019 Feb 15;146(3):dev172684. doi: 10.1242/dev.172684 (PMC6382010; doi:10.1242/dev.172684)
Supplement: Supplementary information [file develop-146-172684-s1.pdf]

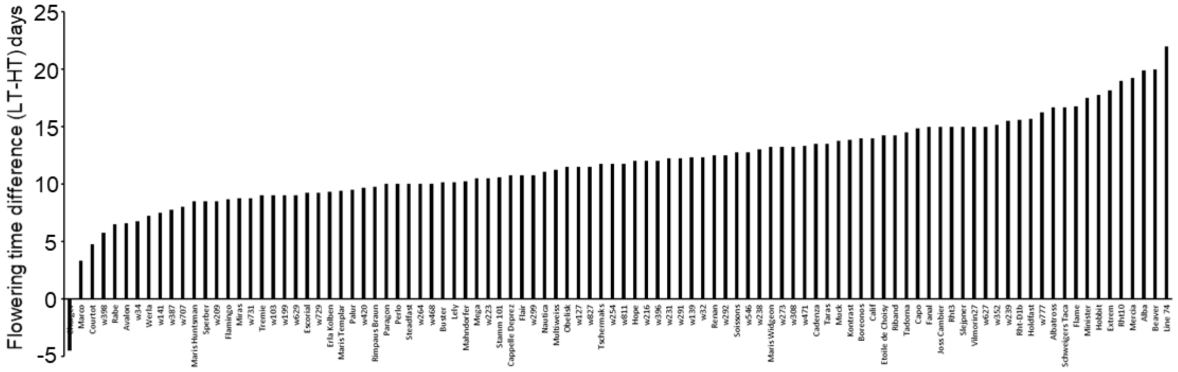

Figure S1. Cultivar analysis of flowering time responses to different temperatures. Difference in flowering time in days between LT (18°C: 13°C) and HT (24°C: 19°C) for landrace and elite wheat cultivars.

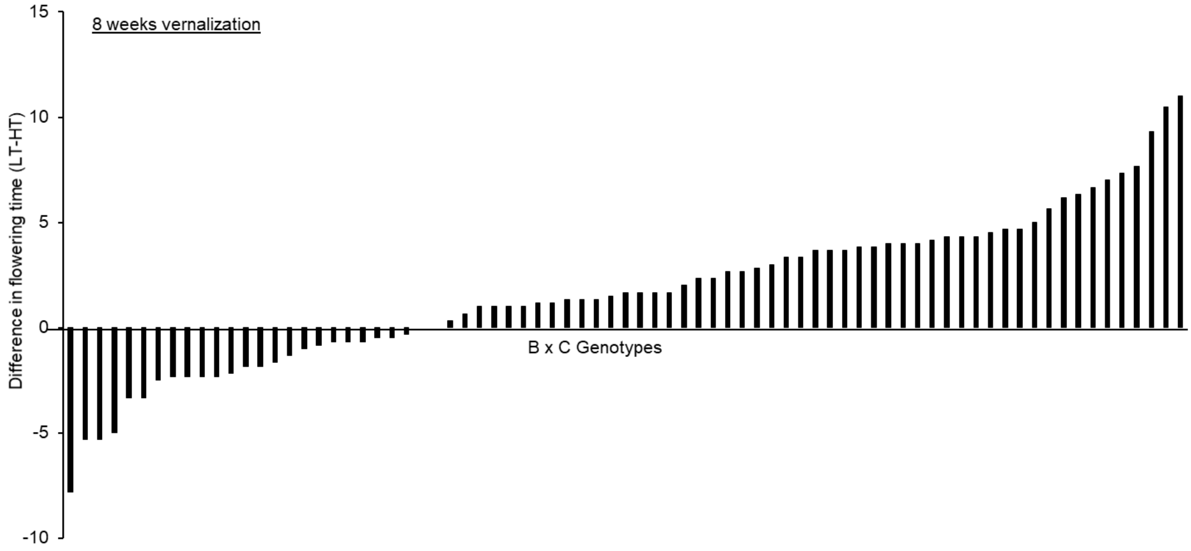

Figure S2. Buster x Charger population analysis of flowering time responses to different temperatures. Difference in flowering time in days between LT (18°C: 13°C) and HT (24°C: 19°C) for Buster x Charger (B x C) doubled haploid population following 8 weeks vernalization. Five genotypes did not reach flowering under the HT and have not been included in this graph. For each temperature condition N = 3 plants per genotype.

A.

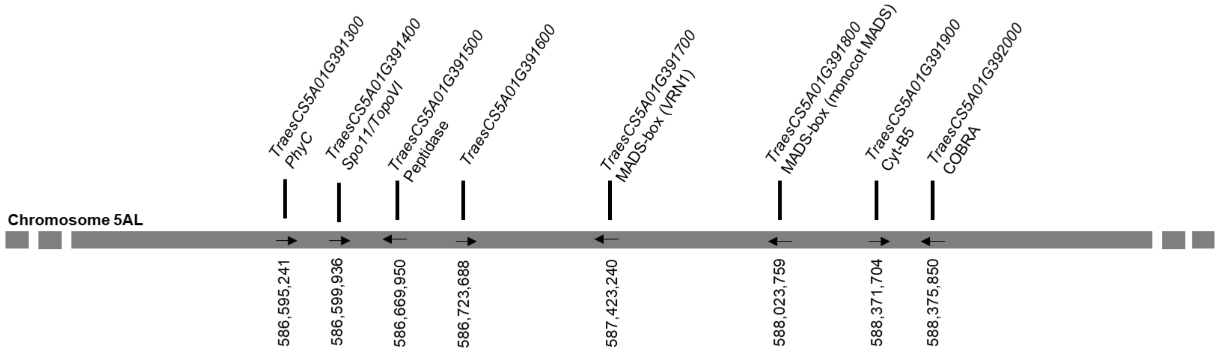

B.

Gene/marker name	Gene/Function	Position on IWGSC	Marker sequence
TraesCS5A01G391300	*PhyC*	586,595,241	
BoBWhite_c471_2245		586,599,596	CTGTTGTTAGGTTTTTAGTGTAATTTTAACACGGCAACTCTACGAGTGCG[C/T]GATGAAATAAAATTAAAATATGTGC TTGTGTTCTTCGATCMATTCTCAGG
TraesCS5A01G391400	*Spo11/TopoVI*	586,599,936	
TraesCS5A01G391500	*Peptidase*	586,669,950	
TraesCS5A01G391600		586,723,688	
TraesCS5A01G391700	*MADS-box (VRN1)*	587,423,240	
TraesCS5A01G391800	*MADS-box (monocot MADS)*	588,023,759	
TraesCS5A01G391900	*Cyt-B5*	588,371,704	
TraesCS5A01G392000	*COBRA*	588,375,850	
TraesCS5A01G392100		588,416,260	
TraesCS5A01G392200		588,454,622	
Tdurum_contig10843_745/TraesCS5A01G402100LC	*proteolysis*	588,496,995	ACCATGATGTGGTTCATGGCATGGCAGGCATCTCCTGAGATGCACAGTTG[A/G]ttcggcaatgctaaaccaacccaat tttttttgctgatgatctctgctg
Ku_c21002_1075		588,496,995	TGCACTAGTTAGTAGTTACAGAAAGTGCTACTGAAAGAACTACTCCCTCC[G/T]TTCACTTTTGTAAGTCATTTCAGAC AACTCAAAATGGGATGTTTTGTACA
TraesCS5A01G392300	*transmembrane transport*	588,548,991	
TraesCS5A01G392400	*protein binding WD-40*	588,553,972	
TraesCS5A01G392500	*protein phosphorylation*	588,731,012	
TraesCS5A01G392600	*ABC-transporter*	588,740,030	
TraesCS5A01G392700	*ABC-transporter*	588,756,468	
TraesCS5A01G392800		588,844,824	
TraesCS5A01G392900	*ribosomal*	588,852,154	
Kukri_c6669_145		588,872,215	ctgcacggcatagcggtgccgctgaaatggccgtcgctcgaagggactccRaggggaagtgctgctgcgcctagcatcgctgtacgtgacaaccttcccct
TraesCS5A01G393000	*protein phosphorylation*	588,873,342	

C.

Gene	Chromosome	IWGSC name
VRN-A1	5AL	*TraesCS5A01G391700*
VRN-B1	5BL	*TraesCS5B01G396600*
VRN-D1	5DL	*TraesCS5D01G401500*

Figure S3. *VRN-A1* and surrounding genes on chromosome 5A.

A) Scheme of the relative (not to scale) positions on Chromosome 5A of *VRN1* and neighbouring genes, with the position of the start of each given sequence from the IWGSC RefSeq v1.0 and the direction of the gene. **B)** The position of the genes and the nearest markers (highlighted in yellow) used in the identification of the QTL position. The markers used have been identified through analysis of Buster x Charger and Avalon x Cadenza maps for the 5A region. The order of the markers used in these maps and the order of the markers on the IWGSC RefSeq v1.0 are different. **C)** Table summarizes the gene identities for *VRN-A1* and homeologues on the B and D genome.

Development • Supplementary information

A

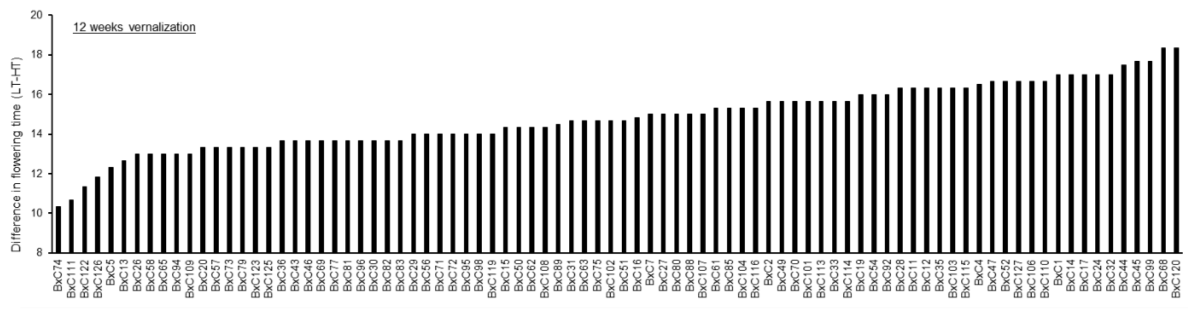

B

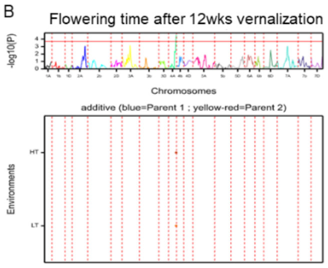

C

Experiment	Vernalization	Condition	Locus name	Position	Linkage group	% Explained var	Allele effect
JIC_8WK	8 weeks	HT	Tdurum_contig1 0843_745	181.4	5A	21.5	Charger
JIC_8WK	8 weeks	HT plus late	Tdurum_contig1 0843_745	181.4	5A	16.6	Charger
JIC_8WK	8 weeks	HT plus late	BS00040403_51	70.2	5A	13.9	Buster
JIC_8WK	8 weeks	HT plus late	Excalibur_rep_c1 13261_400	22.7	4B	11.7	Buster
JIC_12WK	12 weeks	HT	wsnp_Ex_c6514 _11307200	102.9	4A		Charger
JIC_12WK	12 weeks	LT	wsnp_Ex_c6514 _11307200	102.9	4A		Charger

Figure S4. Buster x Charger population analysis of flowering time responses to different temperatures following 12 weeks vernalization. A) Difference in flowering time in days between LT (18°C: 13°C) and HT (24°C: 19°C) for Buster x Charger (B x C) doubled haploid population following 12 weeks vernalization. For each temperature condition 3 plants were grown per genotype. **B)** QTL analysis of doubled haploid Buster x Charger population following 12 weeks of vernalization and then growth under either 18°C: 13°C or 24°C: 19°C; Buster (blue) and Charger (orange) dots on QTL plot. **C)** QTL analysis of half-ear emergence for the Buster x Charger population. The table includes two analysis results from the JIC_8WK vernalization experiment with HT plus late being the analysis which included all genotypes and HT being the analysis when the extremely late/not flowering genotypes were excluded.

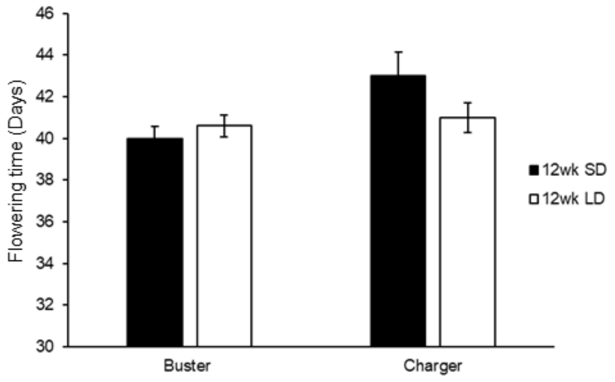

Figure S5. Flowering time of Buster and Charger following vernalization under different photoperiods. Days to flowering for Buster and Charger cultivars following 12 weeks vernalization under short-day (SD) photoperiods (black bars), N=3, and long-day (LD) photoperiods (white bars), N=5. Error is standard error of the mean.

Development • Supplementary information

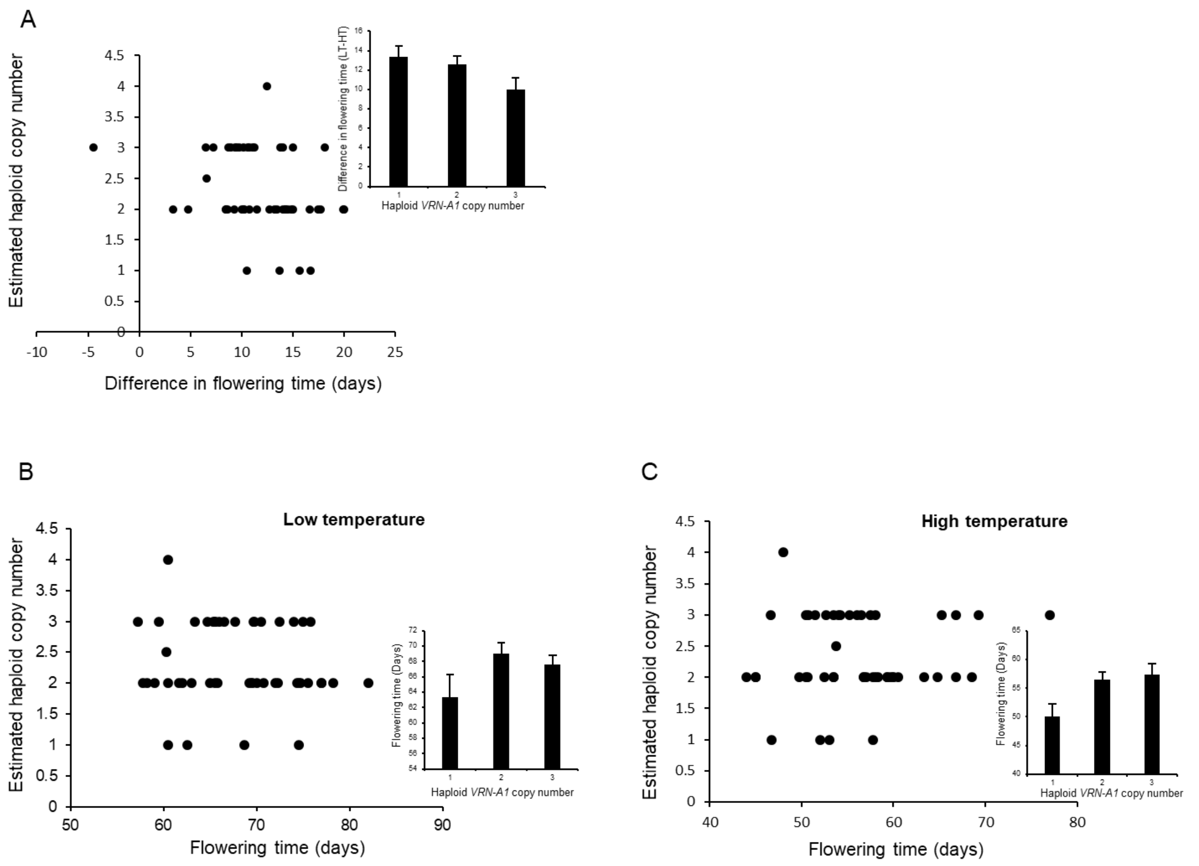

Figure S6. Analysis of flowering time and haploid copy number of *VRN-A1*. A) Difference in flowering time between LT and HT conditions for screen cultivars compared with haploid copy number of *VRN-A1*. **B - C)** Flowering time under **B)** low temperatures (LT) or **C)** high temperatures (HT), compared with haploid copy number with inset showing the average flowering time for each haploid copy number group. Error is standard error of the mean.

Development • Supplementary information

Cultivar name	Country of Origin	Seed collection/source	Type
#74	Experimental cultivar	JIC	s
Alba	Netherlands	Gediflux/JIC	w
Albatross	Belgium	Gediflux/JIC	w
Avalon	Great Britain	JIC	w
Beaver	Great Britain	Gediflux/JIC	w
Boreonos	West Germany	Gediflux/JIC	w
Buster	Great Britain	JIC	w
Cadenza	Great Britain	JIC	s
Calif	Germany	Gediflux/JIC	w
Capo	Austria	Gediflux/JIC	w
Cappelle Deprez	France, Great Britain	Gediflux/JIC	w
Charger	Great Britain	JIC	w
Courtot	France	Gediflux/JIC	w
Erla Kolben	Austria	Gediflux/JIC	w
Escorial	Belgium	Gediflux/JIC	w
Etoile de Choisy	France	Gediflux/JIC	w
Extrem	Austria	Gediflux/JIC	w
Fanal	West Germany	Gediflux/JIC	w
Flair	Germany	Gediflux/JIC	w
Flame	Great Britain	Gediflux/JIC	w
Flamingo	Great Britain, Netherlands	Gediflux/JIC	w
Florida	Denmark	Gediflux/JIC	w
Hobbit	Great Britain	Gediflux/JIC	w
Holdfast	Great Britain	Gediflux/JIC	w
Hope-1	U.S.A.	JIC	s
Joss Cambier	France, Great Britain, Belgium	Gediflux/JIC	w
Kontrast	Germany	Gediflux/JIC	w
Lely	Netherlands	Gediflux/JIC	w
Mahndorfer	East Germany	Gediflux/JIC	w
Marco	Belgium	Gediflux/JIC	w
Maris Huntsman	Great Britain, France, Belgium	Gediflux/JIC	w
Maris Templar	Great Britain	Gediflux/JIC	w
Maris Widgeon	Great Britain	Gediflux/JIC	w
Mega	Great Britain	Gediflux/JIC	w
Minister	Great Britain, Netherlands	Gediflux/JIC	w
Miras	West Germany	Gediflux/JIC	w
Muck	West Germany	Gediflux/JIC	w
Multiweiss	Austria	Gediflux/JIC	w
Nautica	Netherlands	Gediflux/JIC	w
Obelisk	Germany, Denmark	Gediflux/JIC	w
Palur	Germany	Gediflux/JIC	w
Paragon	Great Britain	JIC	s
Perlo	Austria	Gediflux/JIC	w
Rabe	East Germany	Gediflux/JIC	w
Renan	Austria, France	Gediflux/JIC	w
Riband	Great Britain	Gediflux/JIC	w
Rimpaus Braun	East and West Germany	Gediflux/JIC	w
Schweigers Taca	Austria	Gediflux/JIC	w
Slejpner	Sweden, Great Britain, Belgium, Denmark	Gediflux/JIC	w
Soissons	France	Gediflux/JIC	w
Sperber	East Germany	Gediflux/JIC	w
Stamm 101	Austria	Gediflux/JIC	w
Starke2	Sweden	Gediflux/JIC	w
Steadfast	Great Britain	Gediflux/JIC	w
Tadorna	Netherlands	Gediflux/JIC	w
Taras	West Germany	Gediflux/JIC	w
Tremie	France	Gediflux/JIC	w
Tschermaks	Austria	Gediflux/JIC	w
Vilmorin27	France, Great Britain	Gediflux/JIC	w
Werla	East Germany	Gediflux/JIC	w
w103	Italy	Watkins/JIC	s
w127	India	Watkins/JIC	s
w139	France	Watkins/JIC	s
w141	China	Watkins/JIC	s
w160	Spain	Watkins/JIC	s
w199	India	Watkins/JIC	s
w209	Egypt	Watkins/JIC	s
w216	Morocco	Watkins/JIC	s
w223	Burma	Watkins/JIC	s
w231	Hungary	Watkins/JIC	
w238	Iran	Watkins/JIC	s
w239	Spain	Watkins/JIC	s
w254	Morocco	Watkins/JIC	s
w264	Canary Islands	Watkins/JIC	s
w273	Spain	Watkins/JIC	s
w291	Cyprus	Watkins/JIC	s
w292	Cyprus	Watkins/JIC	s
w299	Turkey	Watkins/JIC	s
w300	Turkey	Watkins/JIC	s
w308	Iran	Watkins/JIC	s
w32	India	Watkins/JIC	s
w34	India	Watkins/JIC	s
w349	Bulgaria	Watkins/JIC	s
w352	Former Yugoslavia	Watkins/JIC	s
w387	Spain	Watkins/JIC	s
w396	Portugal	Watkins/JIC	s
w398	Palestine	Watkins/JIC	s
w420	India	Watkins/JIC	s
w468	Afghanistan	Watkins/JIC	s
w471	Afghanistan	Watkins/JIC	s
w546	Spain	Watkins/JIC	s
w627	Iran	Watkins/JIC	s
w629	Iran	Watkins/JIC	s
w707	India	Watkins/JIC	s
w729	Iran	Watkins/JIC	s
w731	India	Watkins/JIC	s
w777	Finland	Watkins/JIC	s
w811	Tunisia	Watkins/JIC	s
w827	China	Watkins/JIC	s
Mercia	Experimental cultivar	JIC	w
rht3	Experimental cultivar	JIC	w
rht10	Experimental cultivar	JIC	w
rhtd1b	Experimental cultivar	JIC	w

Table S1. Genotypes, origin and growth habit temperature of cultivars used in ambient screen. Genotypes used in the ambient temperature screen. The table details genotype name, country of origin, germplasm collection and growth habit (w = winter, s = spring).

Table S2. Buster x Charger population genetic map

Buster x Charger population map, as described in Materials and Methods. Coding for the genotypes 1 = Buster and 2 = Charger. Due to the size of this file it has been submitted as a separate excel file.

Click here to download Table S2

A.

VRN-A1						
**Cultivar**	**allelic composition**	**Promoter insertion**	**Intron 1 deletion**	**exon 4 CNV**	**exon 7**	**Consequence**
Buster	*vrn-A1 vrn-B1 vrn-D1*	Winter	None	C,T	T	Winter
Charger	*vrn-A1 vrn-B1 vrn-D1*	Winter	None	C,T,T	T	Winter
Hereward	*vrn-A1 vrn-B1 vrn-D1*	Winter	None	C,T,T	T	Winter
Malacca	*vrn-A1 vrn-B1 vrn-D1*	Winter	None	C,T	T	Winter
Wichita	*vrn-A1 vrn-B1 vrn-D1*	Winter	None	T (Vrn-A1w)		Winter
Jagger	*vrn-A1 vrn-B1 vrn-D1*	Winter	None	C (Vrn-A1v)		Winter
Triple Dirk B	*vrn-A1 Vrn-B1 vrn-D1*	Winter	B	C		Spring
Triple Dirk C	*vrn-A1 vrn-B1 vrn-D1*	Winter	None	C	C	Winter
Triple Dirk D	*Vrn-A1 vrn-B1 vrn-D1*	A insertion	None	C	C	Spring
Triple Dirk E	*vrn-A1 vrn-B1 Vrn-D1*	Winter	D	C	C	Spring
Chinese Spring	vrn-A1 vrn-B1 Vrn-D1	Winter	D	C	C	Spring
Paragon	vrn-A1 Vrn-B1 vrn-D1	Winter	B			Spring

B.

VRN-A1										***VRN-D4***
**SNP/ Indel position**	10228	10428	10670	10959	11054	11292	11322	11339		10446
		exon4	insertion	deletion	exon7		exon8	exon8		exon 4
**Buster**	a	C(8) t(2)	t	t	a	C(2) t(5)	C(5) t(3)	C(5) t(3)		no SNP
**Charger**	a	C(33) t(47)	t	t	a	C(9) t(1)	C(1) t(2)	C(1) t(4)		no SNP
**Avalon**	a	C(42) t(29)	t	t	a	C(2) t(1)	C(1)	C(1)		no SNP
**Badger**	a	C(29) t(46)	t	t	a	-	C(3) t(9)	C(3) t(7)		no SNP
**Spark**	G	C(81)	t(4)	-	G	-	C(2) t(6)	C(2) t(7)		no SNP
**Rialto**	a	C(51) t(27)	t	t	a	C(9) t(2)	C(3) t(7)	C(3) t(8)		no SNP

Table S3. *VRN-A1* genotypes. A) Summary table of the known *VRN-A1* genotypes with a focus on allelic variations which alter habit and the exon 4 and 7 SNPs. Information collated from Fu et al., 2005, Eagles et al., 2011, Díaz et al., 2012, this study and available sequence on Pubmed.

B) The SNP/ Indel position is determined with the first coding nucleotide corresponding to position 1. Where more than one allelic variant is observed at the same SNP position the read depth from the exome capture is given in brackets.

Development • Supplementary information

A.

ID	Haploid CNV	Exon 4 SNP
Alba	2	long
Albatross	2	long
Avalon	2.5	long
Beaver	2	long
Boreonos	3	-
Buster	2	long
Calif	2	short
Capo	2	long
Cappelle Deprez	2	-
Charger	3	long
Courtot	2	-
Ellvis	2	-
Erla Kolben	3	long
Escorial	2	long
Etoile de Choisy	2	long
Extrem	3	long
Fanal	2	long
Flair	3	long
Flame	1	short
Flamingo	2	long
Florida	3	long
Hobbit	2	long
Holdfast	1	short
Joss Cambier	3	long
Kontrast	3	-
Lely	3	long
Mahndorfer	2	long
Marco	2	-
Maris Huntsman	2	long
Maris Widgeon	2	short
Mega	1	short
Minister	2	long
Miras	3	long
Muck	1	short
Multiweiss	3	long
Nautica	3	long
Obelisk	2	long
Palur	3	long
Rabe	3	long
Renan	4	long
Riband	2	-
Rimpaus Braun	3	long
Soissons	2	long
Sperber	2	long
Stamm 101	3	long
Starke2	2	long
Steadfast	2	long
Tadorna	2	long
Taras	2	long
Toborzo	2	-
Tschermaks	3	-
Ukrainka	3	-
Verbunkos	2	-
Werla	3	long

B.

Copy Number	Long Vern Allele	Short Vern Allele
4	1	0
3	15	0
2.5	1	0
2	20	2
1	0	4

C.

Cultivar	Haploid copy number	Time to flowering (Days)	
		HT	LT
B x C 98 216D	3	did not flower in exp	52.5
B x C 107 242A	3	did not flower in exp	53
B X C 27 74A	3	did not flower in exp	56
B x C 126 377C	2	50	50
B x C 1 27A	2	48.6	48
B x C 30 75G	2	46	50.5

Table S4. Taqman estimates of haploid *VRN-A1* copy number. A) *VRN-A1* haploid copy number estimates and indication of the Exon 4 SNP type. **B)** Summary table of cultivar *VRN-A1* haploid copy number. **C)** *VRN-A1* haploid copy number estimates for six examples from the Buster x Charger doubled haploid population with flowering time under the low and high temperature conditions used in the screen.
